# Centrifugally Spun Binder-Free N, S-Doped Ge@PCNF Anodes for Li-Ion and Na-Ion Batteries

**DOI:** 10.1021/acsomega.3c00990

**Published:** 2023-05-03

**Authors:** Meltem Yanilmaz, Göktuğ Cihanbeyoğlu, Juran Kim

**Affiliations:** † Nanoscience and Nanoengineering, Istanbul Technical University, Istanbul 34469, Turkey; ‡ Department of Textile Engineering, Istanbul Technical University, Istanbul 34469, Turkey; § Advanced Textile R&D Department, Korea Institute of Industrial Technology (KITECH), Ansan 15588, Republic of Korea

## Abstract

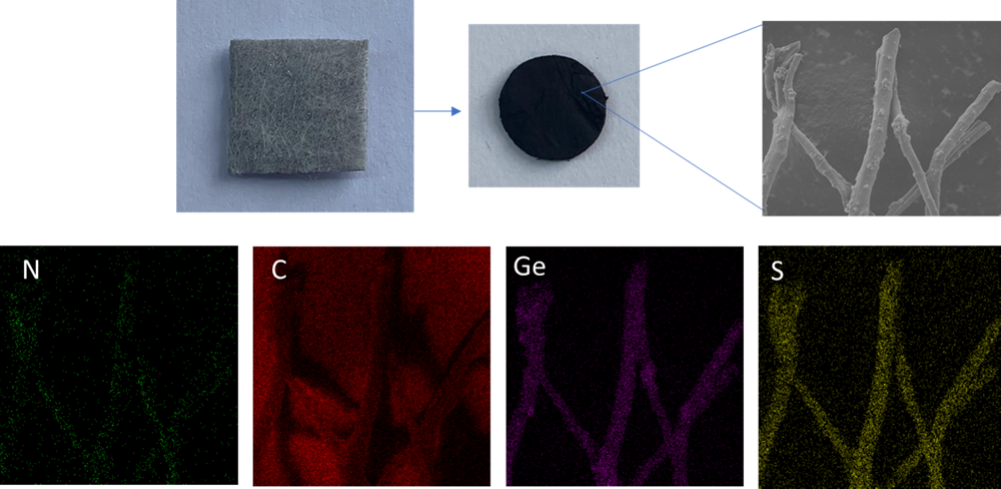

Germanium has a high theoretical capacity as an anode material for sodium-ion batteries. However, germanium suffers from large capacity losses during cycling because of the large volume change and loss of electronic conductivity. A facile way to prepare germanium anodes is critically needed for next-generation electrode materials. Herein, centrifugally spun binder-free N, S-doped germanium@ porous carbon nanofiber (N, S-doped Ge@ PCNFs) anodes first were synthesized using a fast, safe, and scalable centrifugal spinning followed by heat treatment and N, S doping. The morphology and structure of the resultant N, S-doped Ge@ PCNFs were investigated by scanning electron microscopy, transmission electron microscopy, energy-dispersive X-ray mapping, Raman spectroscopy, and X-ray diffraction, while electrochemical performance of N, S-doped Ge@ PCNFs was studied using galvanostatic charge–discharge tests. The results demonstrate that a nanostructured Ge homogeneously distributed on tubular structured porous carbon nanofibers. Moreover, N, S doping via thiourea treatment is beneficial for lithium- and sodium-ion kinetics. While interconnected PCNFs buffered volume change and provided fast diffusion channels for Li ions and Na ions, N, S-doped PCNFs further improved electronic conductivity and thus led to higher reversible capacity with better cycling performance. When investigated as an anode for lithium-ion and sodium-ion batteries, high reversible capacities of 636 and 443 mAhg^–1^, respectively, were obtained in 200 cycles with good cycling stability. Centrifugally spun binder-free N, S-doped Ge@ PCNFs delivered a capacity of 300 mAhg^–1^ at a high current density of 1 A g^–1^, indicating their great potential as an anode material for high-performance sodium-ion batteries.

## 1 Introduction

Renewable energy research studies have been intensively increased to address the problems arising from the massive usage of fossil fuels such as serious environmental problems. Burning fossil fuels emits carbon dioxide and leads to global warming. It is possible to mitigate negative effects of fossil-based economy, reduce the dependence of fossil fuels, and decrease the emission of greenhouse gases by using renewable energy sources. Development of energy storage systems which are capable of storing electrical energy harvested from renewable sources is important. In this regard, secondary batteries have been the center of the attention due to the efficient storage and delivery of the electrical energy. Lithium-ion batteries (LIBs) have been used in a wide range of portable electronics and electric vehicles (EVs) owing to their long cycle life and large energy-conversion efficiency. Moreover, sodium-ion batteries (SIBs) have attracted great attention because of high abundance and low cost of sodium resources. SIBs present opportunities for potential applications in large-scale grid energy storage.^1,2^

Considering increasing demand and high requirements of new applications including EVs, anode materials with high capacity and low-cost preparation methods need to be developed.^3−6^ Germanium (Ge) is a promising anode material because of its fast Li^+^ diffusivity, good electronic conductivity (small band gap of 0.6 eV), and high theoretical capacity (1600 mAh g^–1^ in LIBs and 369 mAh/g in SIBs). However, the large volume expansion, pulverization, and electrical disconnects between the active materials and the electrode framework lead to poor cycling performance.^7,8^ Designing carbon composite anode materials could confine the volume expansion of Ge and maintains the good integrity of the anode which could lead to long cycle life. Carbon nanofibers (CNFs) provide excellent electrical connection, high lithium-ion transport kinetics, and facile strain relaxation. Moreover, by tailoring their surface texture, diameter, and porosity, many active sites could be created and volume changes occurred during intercalation or alloying could be buffered.^9−11^ Nanostructured Ge dispersed on fibrous carbons is a facile strategy to relieve the stress caused by volume expansion. Moreover, specific capacity of pure CNFs can be further enhanced by constructing pores in the structure. Highly porous CNFs not only buffer the volume changes but also limit direct exposure of germanium particles to the electrolyte. High electrical conductivity of carbon provides essential transport channels for both electrons and ions while a highly porous structure shortens electron and ion transport lengths, which enhances cell kinetics and thus rate capability and power density. Moreover, heteroatom doping could further increase defects and active sites in the anode structures. Heteroatom doping can generate more active sites on the surface and enhance electronic conductivity, leading to high electrochemical activity and reversible specific capacity. Sulfur (S), phosphorus (P), boron (B), and nitrogen (N) have attracted great attention and have been highly investigated. Doped N or B atoms substitute for carbon atoms in the hexagonal lattice; on the other hand, S or P atoms are placed between the lattices.^12,13^

Electrospinning is the most commonly used technique to produce nanofibers. However, excessive solvent usage, high voltage needed during production, and low production rates limit the commercialization of this technique. Centrifugal spinning allows producing nanofibers at very high production rates without applying high voltage. Furthermore, this technique has the merits of low cost and environmental friendliness due to low consumption of toxic solvents.^14,15^

Recently, different approaches have been reported to prepare Ge/carbon anodes such as solution-based, thermal evaporation, arc-discharge, and chemical vapor deposition methods. Among them, nanostructured one-dimensional (1D) hybrid electrodes are promising considering the benefits of 1D nanostructured carbon electrodes including buffering against volume expansion, high interfacial stability, high conductivity, short electron and ion transport channels, and hybrid structure; however achieving uniform distribution of active materials is challenging, and pulverization and aggregation of active materials lead to capacity fading.^7,16,17^ For example, Xie et al.^16^ reported GeO_2_-included carbon nanofibers and a reversible capacity of over 500 mAh/g was reported in the first 60 cycles; however, a long-term cycling study was not reported. Youn et al.^18^ used carbon spheres to buffer the volume change and reduce the aggregation of Ge, and a reversible capacity of around 300 mAh/g was reported in 200 cycles for Ge/C anodes. Nanostructure, uniform particle distribution, and a highly conductive carbon network are essential to develop long-lasting electrodes with high performance; however, preparation of Ge-based composites with these features remains challenging.^19^ It is critical to develop a fast and facile technique to create 1D nanostructured hybrid electrodes with uniform distribution. Herein, Ge-included highly porous carbon nanofibers with a tubular structure were fabricated for the first time via combining fast, safe, and environmentally friendly centrifugal spinning, heat treatment, and N, S doping and then used as a binder-free anode in Li-ion and Na-ion batteries. The excellent electrochemical properties can be attributed to the homogeneous distribution of germanium, high contact area between the electrode and the electrolyte owing to the highly porous tubular structure of carbon nanofibers, and the improved conductivity via N, S doping.

## 2 Experimental Section

Polyacrylonitrile (PAN), polystyrene (PS), *N*, *N*-dimethylformamide, thiourea, lithium hexafluorophosphate (LiPF_6_), dimethyl carbonate, diethyl carbonate, sodium perchloride (NaClO_4_), ethylene carbonate (EC), propylene carbonate (PC), fluorinated ethylene carbonate (FEC), sodium metal, and Li foil were purchased from Sigma-Aldrich. Glass fibers were purchased from Whatman. PAN/PS nanofibers were prepared by centrifugal spinning; a 10 wt.% PAN/PS solution was fed into the spinneret, and a rotational speed of 4000 rpm was applied. The spinneret-to-collector distance was set at 10 cm. To obtain Ge@PCNFs, a certain amount of Ge was included in PAN/PS solution before centrifugal spinning, and Ge-included PAN/PS nanofibers were stabilized at 280 °C for 3 h and carbonized at 800 °C under nitrogen. In addition, N, S-doped germanium@porous carbon nanofibers (N, S-doped Ge@ PCNFs) were prepared by heating Ge@ PCNFs with thiourea with a mass ratio of 1:5 in a nitrogen atmosphere at 500 °C for 2 h with a ramp rate of 10 °C min^–1^.

SEM (Zeiss Sigma 300, Germany) was used to study the morphology of N, S-doped Ge@ PCNFs. FE-SEM (QUANTA FEG 250) was used for energy-dispersive X-ray (EDX) mapping. X-ray diffraction (XRD) (PANalytical Empyrean, UK) with a step of 0.01 and a speed of 4° per minute and Raman spectroscopy (WITech alpha 300R, Germany) was used to characterize the structure. The composition of N, S-doped Ge@ PCNFs was determined by thermogravimetric analysis (TGA, Hitachi, Japan). The sample was heated at a rate of 10 °C/min from room temperature to 900 °C in air.

As-prepared centrifugally spun binder-free N, S-doped Ge@ PCNFs were used directly as anodes in Li-ion and Na-ion cells. The electrochemical performance was measured with CR2032 type coin cells. The centrifugally spun binder-free N, S-doped Ge@ PCNFs electrodes were cut into a 12 mm diameter disk and then used directly as a working electrode without additional metal current collectors, conductive additives, or polymer binders. In lithium-ion cells, the counter/reference electrode was a lithium foil and the electrolyte was 1 M LiPF_6_ in ethylene carbonate/dimethyl carbonate/diethyl carbonate. A polypropylene separator was used in the cells. In Na-ion cells, the counter/reference electrode was a sodium foil freshly prepared using sodium metal (Na, Sigma), the glass fiber film (Whatman GF/D) acted as a separator, and the electrolyte was the solution of 1 M NaClO_4_ in EC/PC with a volume ratio of 1:1 with 5% FEC. Galvanostatic measurements were performed on the battery testing systems (Neware and Hefa cycler) with a potential range of 0.0–2.5 V (vs Na/Na^+^).

## 3 Results and Discussion

**Figure 1:**
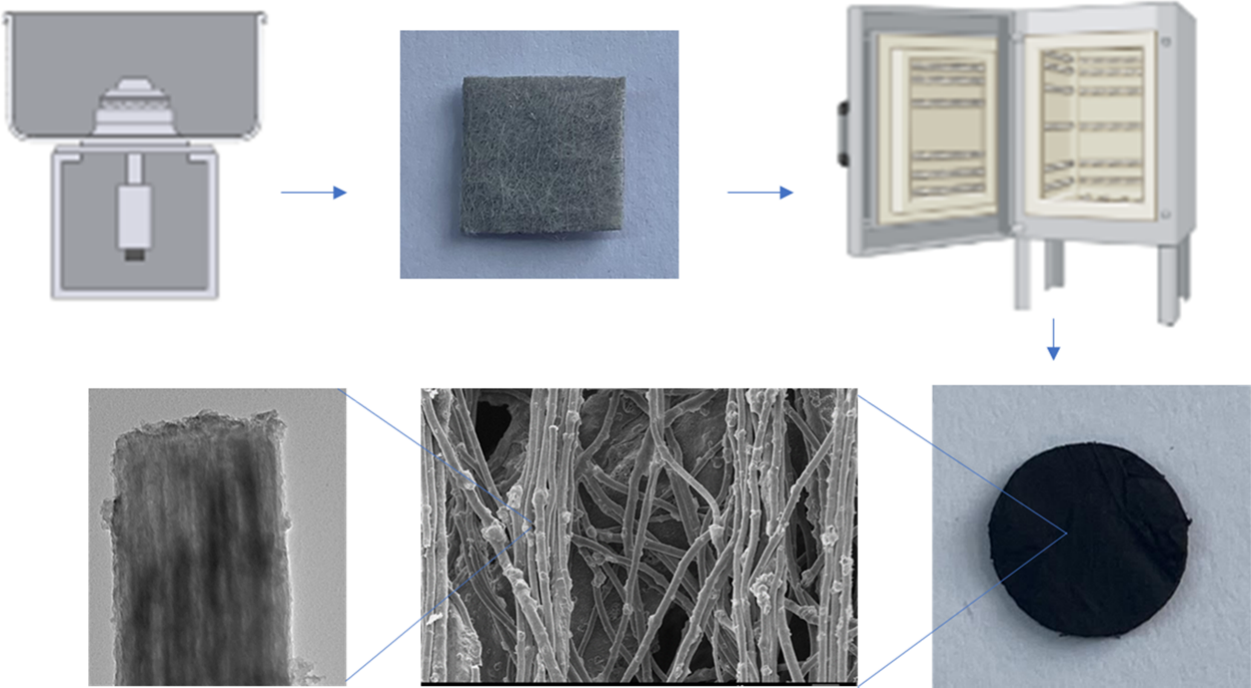
Schematic illustration of preparation of N, S-doped Ge@PCNFs.

Centrifugally spun binder-free N, S-doped Ge@PCNFs were prepared via combining centrifugal spinning, two-step heat treatment, and N, S doping by using thiourea as illustrated in Figure 1. After fibrous mats were obtained by centrifugal spinning, heat treatment was applied to obtain Ge@PCNFs. Thiourea treatment at high temperature was used to further improve electronic conductivity and defects of the binder-free electrodes.

**Figure 2:**
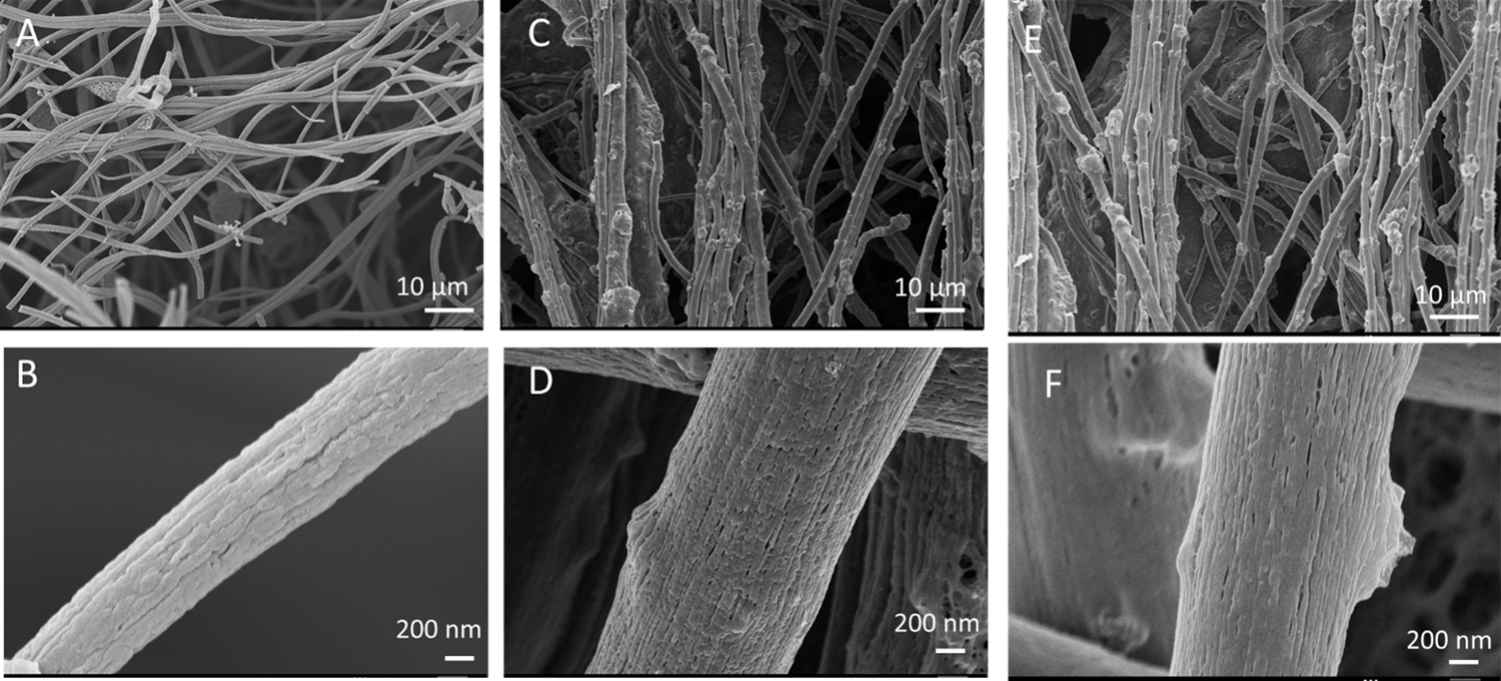
SEM images of PCNFs (A, B), Ge@PCNFs (C, D), and N, S-doped Ge@PCNFs (E, F).

Figure 2 displays SEM images of centrifugally spun PCNFs, Ge@PCNFs, and N, S-doped Ge@ PCNFs. Inclusion of Ge led to larger fiber diameters with a rougher surface. Inclusion of nanoparticles in spinning solution increased the solid content, and thus the diameter of the resultant fibers increased. Wei et al.^20^ prepared Ge/C nanofibers as an anode for LIBs, and a rougher surface with larger fiber diameters was also observed with Ge inclusion. Li et al. investigated the Si effect on Si/C composite nanofibers and reported that Si/C composite nanofibers had a rougher surface compared to pure CNFs.^21^ No significant change was observed with N, S doping on the morphology. Xie et al.^16^ prepared GeO_2_ carbon nanofibers and reported the rough surface with uniform Ge distribution. This morphology led to high reversible capacity with good cycling performance.

**Figure 3:**
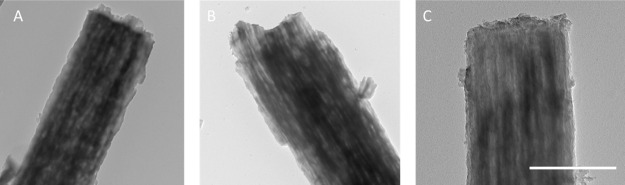
TEM images of PCNFs (A), Ge@PCNFs (B), and N, S-doped Ge@PCNFs (C).

Transmission electron microscopy (TEM) images of PCNFs, Ge@PCNFs, and N, S-doped Ge@ PCNFs are shown in Figure 3. Highly porous tubular structures are observed from TEM images for all studied samples. The high-magnification TEM image for N, S-doped Ge@ PCNFs is presented in Figure S1. Hollow structures provide shorter pathways for ions and electrons and thus improve the kinetics and rate performance. Moreover, more sites for ion intercalation further improve the capacity in porous structures.^22−25^

**Figure 4:**
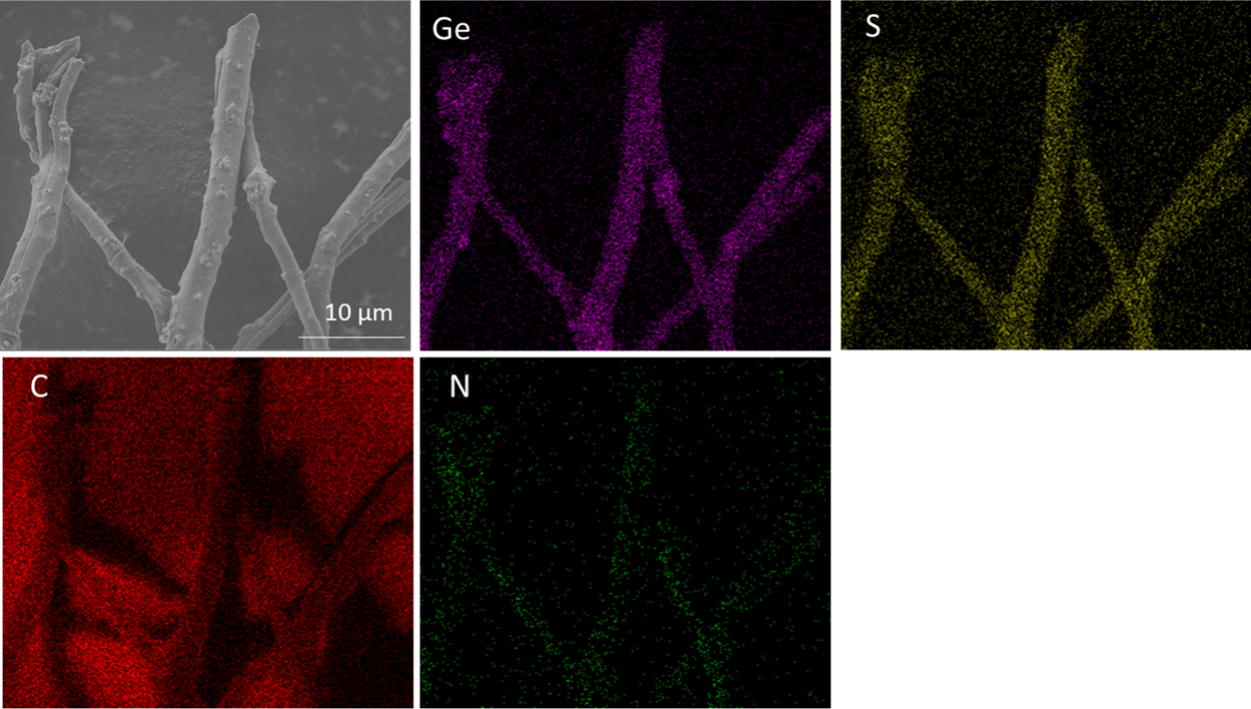
EDX mapping of N, S-doped Ge@PCNFs.

EDX mapping of N, S-doped Ge@ PCNFs is also shown in Figure 4 to present uniform inclusion of Ge in N, S-doped PCNFs. The distributions of N and S are presented as well. The highly uniform Ge is crucial for better cycling performance, and it has been reported that highly dispersed Ge could eliminate particle agglomeration and leads to good cycling performance.^7,23^

**Figure 5:**
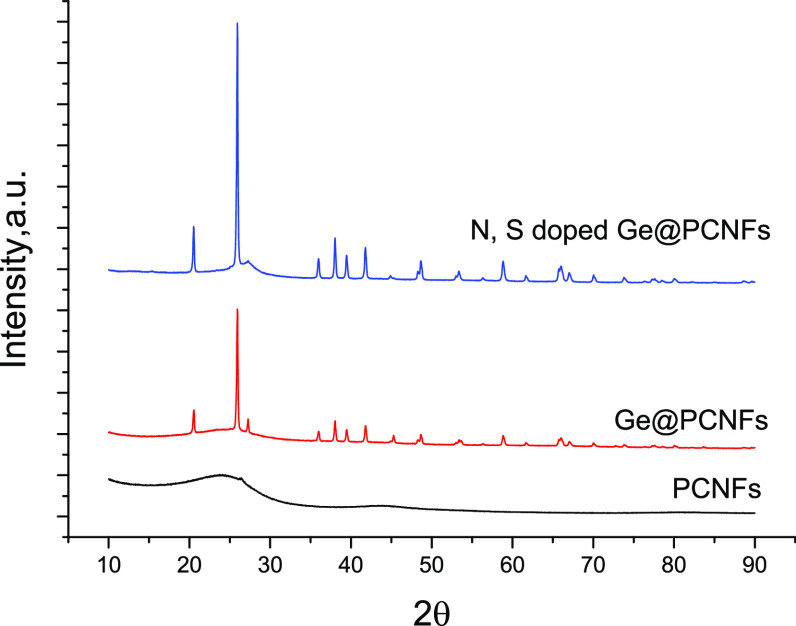
XRD pattern of PCNFs, Ge@PCNFs, and N, S-doped Ge@PCNFs.

The crystalline structure and chemical composition of N, S-doped Ge@ PCNFs were studied by X-ray diffraction (XRD), and the XRD patterns are shown in Figure 5. XRD patterns for CNFs and Ge@ PCNFs are also presented for comparison. In the XRD pattern of PCNFs, a peak at approximately 25° corresponds to the (0 0 2) layers of reflection of the graphite structure, and the wide peak proves the amorphous structure.^26^ XRD patterns of Ge@PCNFs and N, S-doped Ge@PCNFs show strong peaks at around 27°, 45°, 54°, 66°, and 72° which are indexed to (111), (220), (311), (440), and (331) planes of the fcc cubic Ge crystal, respectively (JCPDS card no. 4-545).^8,27^

**Figure 6:**
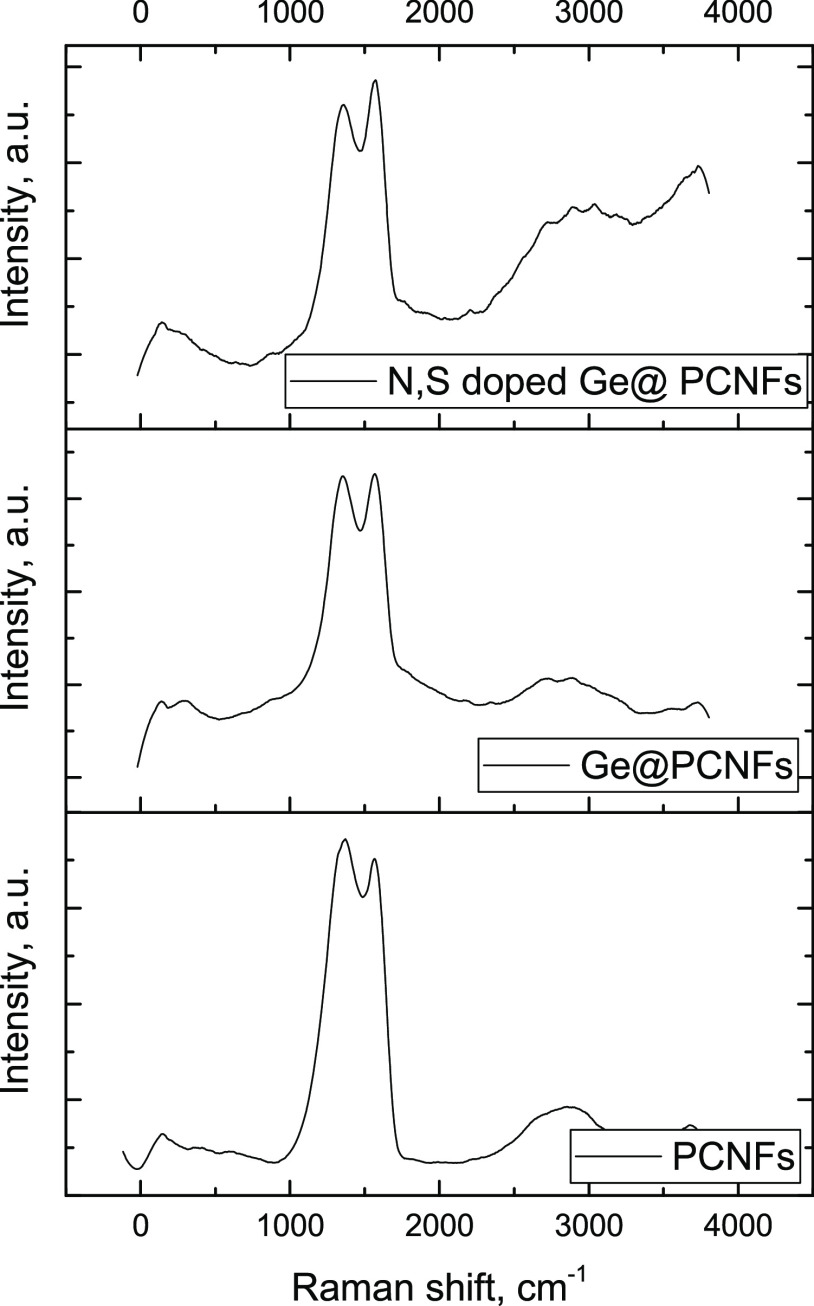
Raman spectra of PCNFs, Ge@PCNFs, and N, S-doped Ge@PCNFs.

**Figure 7:**
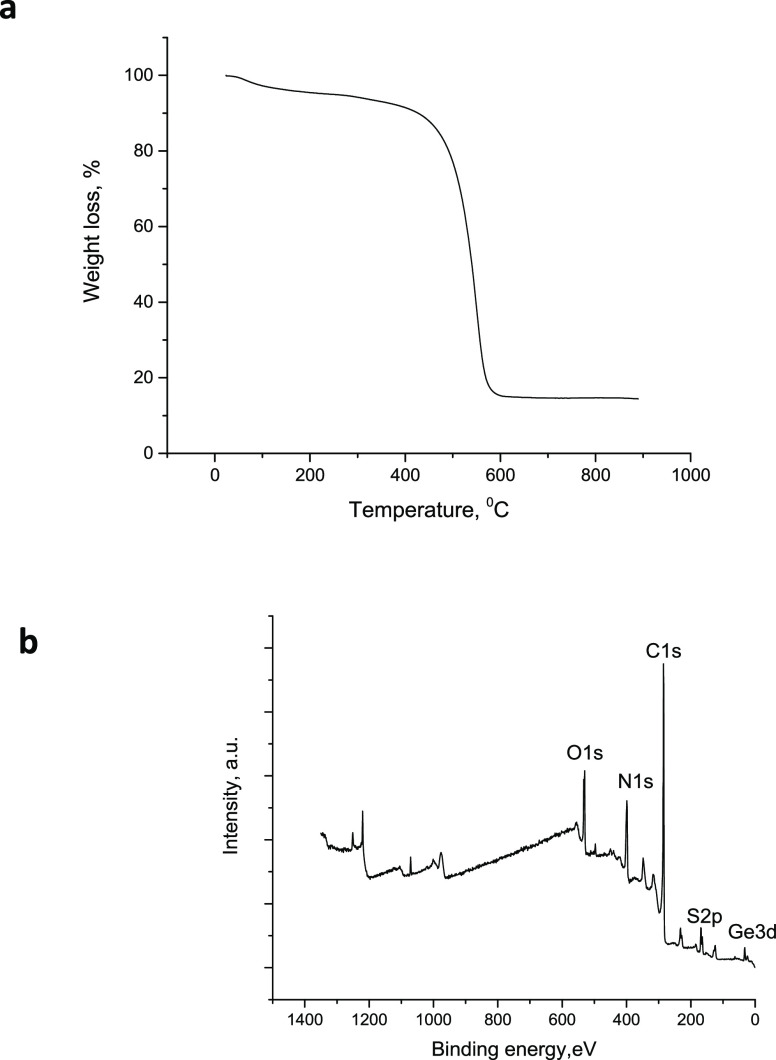
TGA spectra (a) and XPS survey (b) of N, S-doped Ge@PCNFs.

Raman spectra for PCNFs, Ge@ PCNFs, and N, S-doped Ge@ PCNFs are given in Figure 6. All of the prepared samples indicate two main Raman peaks located at around 1400 and 1750 cm^–1^, which correspond to the disordered (D band) and graphitic carbons (G band), respectively. The *I*_D_/*I*_G_ band intensity ratios of N, S-doped Ge@PCNFs and Ge@PCNFs (approximately 0.98) are lower than that of PCNFs (approximately 1.1), indicating the higher degree order for N, S-doped Ge@PCNFs and Ge@PCNFs. These results suggest that a certain amount of Ge atoms can be incorporated into PCNFs and an enhanced graphitic structure would result in increased electrical conductivity, fast ion diffusion, and thus high power density.^5,23,28^ Wei et al.^23^ also fabricated Ge-containing carbon and reported that the intensity ratio of the G band was higher than that of the D band that led to higher conductivity and thus electrochemical properties. The content of germanium in the Ge@ N, S-doped PCNFs was around 18 wt % as determined by TGA as shown in Figure 7a. X-ray photoelectron spectroscopy (XPS) survey for Ge@ N, S-doped PCNFs is presented in Figure 7b, and contents for N and S are 14 and 10%, respectively. High-resolution scans of the S2p spectrum and N1s spectrum are presented in Figure S2. The peaks are approximately at 164 and 169 eV correspond to −C–S–C– bond and −C–SO_*X*_–C– bond. N1s scan also proved that the bonding configurations are graphitic N (398.1 eV) and pyridinic N (401.0 eV). In the pyridinic N structure, nitrogen atoms are at the edge of graphite planes, each of which is bonded to two carbon atoms, whereas nitrogen atoms are incorporated into the carbon network in the graphitic nitrogen.^29−32^ The presence of C–S and C–N peaks confirmed that N and S atoms had been incorporated into the carbon structure.

**Figure 8:**
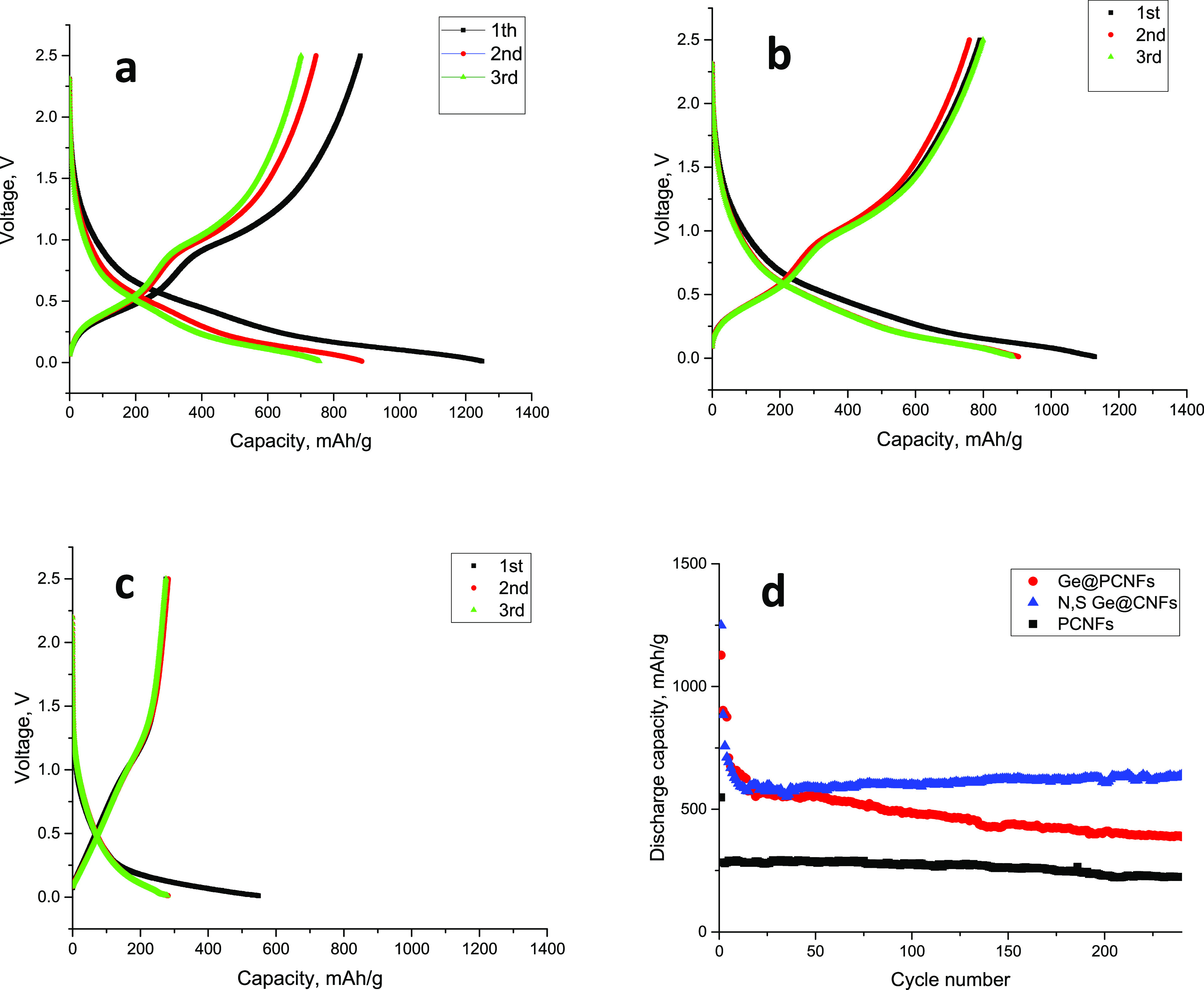
First cycle discharge charge curves of (a) N, S-doped Ge@PCNFs, (b) Ge@PCNFs, and (c) PCNFs and (d) cycling performance of N, S-doped Ge@PCNFs in LIBs.

The electrochemical performance of centrifugally spun binder-free N, S-doped Ge@ PCNFs electrodes is investigated via galvanostatic charge/discharge cycling between 0.01 and 2.5 V using CR2032 coin cells. Figure 8 shows the first three discharge charge curves and cycling performance of centrifugally spun binder-free N, S-doped Ge@ PCNFs electrodes in LIBs. The initial discharge / charge capacities are 1249/ 880, 1128/789, and 548/275 mAh/g respectively for N, S-doped Ge@ PCNFs, Ge@ PCNFs, and PPCNFs at 0.1 A/g. In the first cycles, the large capacity loss can be attributed to the formation of SEI on the surface of the electrode, irreversible Li insertion, and the high surface contact area between the electrode and electrolyte.^8,20,24^ Gao et al.^24^ also prepared Ge-included CNT electrodes and capacity loss in the first cycles ascribed to the SEI and irreversible Li insertion into the electrode. Xie et al.^16^ also reported that irreversible growth of the SEI layer and some side reactions led to the capacity loss in the first cycles for Ge/carbon electrodes. After the first cycles, the reversibility of the capacity was significantly improved. The high capacity of N, S-doped Ge@ PCNFs electrodes could be attributed to many active sites resulting from N, S doping and tubular structure of PCNFs as observed from TEM images.

The reversible capacity of the PCNF electrode was approximately 230 mAh/g while Ge@ PCNFs electrodes delivered capacity over 550 mAh/g in the first 40 cycles; however, a sharp decrease was observed. In 200 cycles, the reversible capacity was around 400 mAh/g for Ge@ PCNFs. Benefiting from N, S doping centrifugally spun binder-free N, S-doped Ge@ PCNFs electrodes delivered the highest capacity with the best cycling performance. In 200 cycles, a reversible capacity of over 630 mAh/g was achieved. The excellent cycling performance could be attributed to enhanced electronic conductivity and more active sites due to N, S doping. Wang et al.^28^ prepared Ge-included CNFs via electrospinning, and a capacity of around 600 mAh/g was achieved; however, only the first 100 cycles was reported and the result was ascribed to nanostructured Ge and excellent electrical conductivity of CNFs.

**Figure 9:**
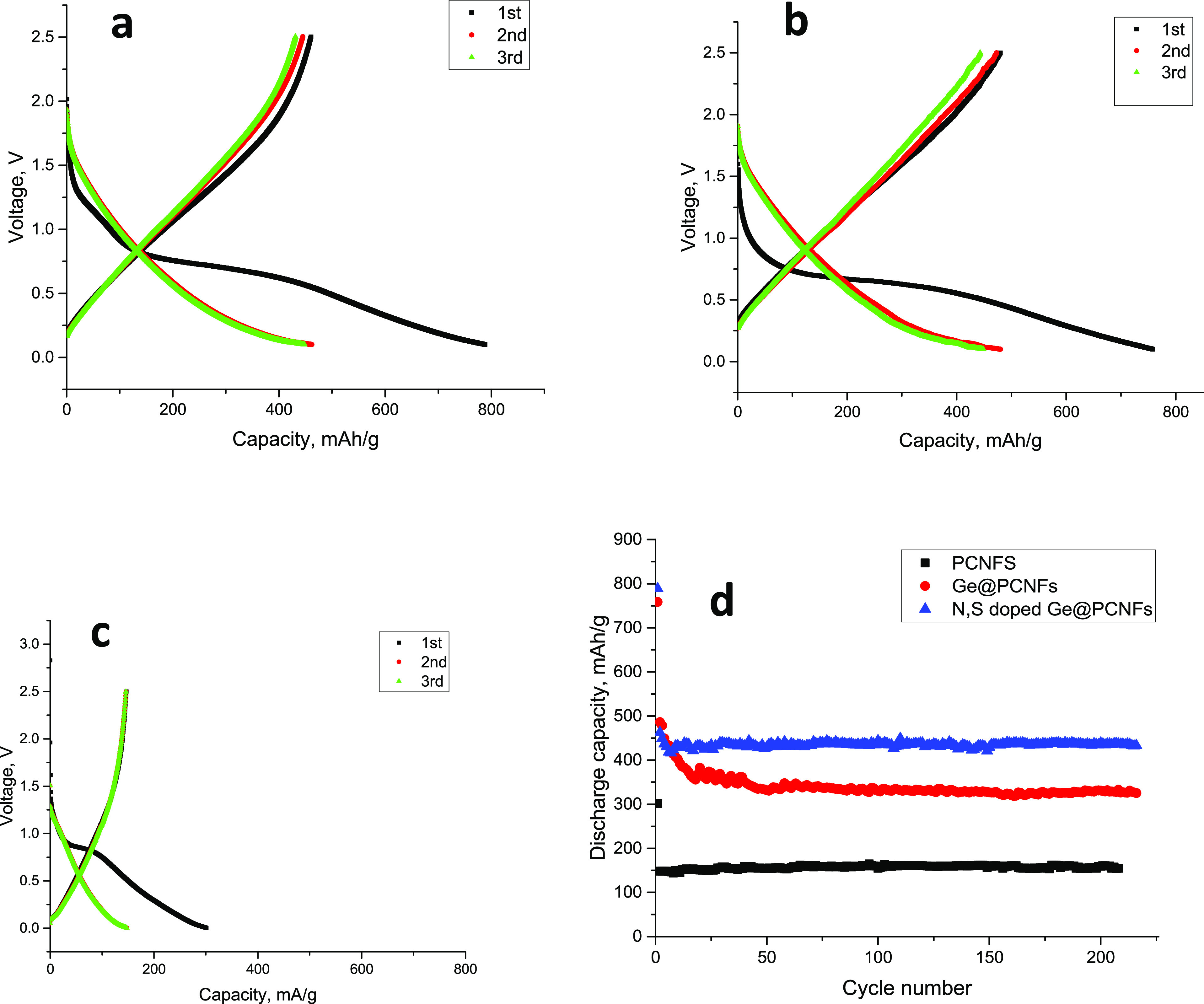
First cycle discharge charge curves of (a) N, S-doped Ge@PCNFs, (b) Ge@PCNFs, and (c) PCNFs and (d) cycling performance of N, S-doped Ge@PCNFs in SIBs.

Figure 9 shows the first three discharge charge curves and cycling performance of centrifugally spun binder-free N, S-doped Ge@ PCNFs electrodes in SIBs. The first discharge charge capacities are 788/460, 758/480, 301, and 146 mAh/g, respectively, for N, S-doped Ge@PCNFs, Ge@PCNFs, and PCNFs. The reversible capacity of PCNF electrodes was approximately 156 mAh/g in 200 cycles, and excellent cycling performance was observed. In comparison, Ge@PCNF electrodes delivered a reversible capacity of around 330 mAh/g while N, S-doped Ge@ PCNFs electrodes had a reversible capacity of approximately 446 mAh/g in 200 cycles with excellent cycling performance. Stable cycling performance was also observed from N-doped carbon by Vadahanambi et al.,^33^ and the result was ascribed to a larger electrolyte-accessible surface area and more defects created by substitution of N atoms in the carbon structure. Li et al.^34^ prepared Ge-containing hollow carbon electrodes, and a reversible capacity of approximately 360 mAh/g with good cycling performance was presented, and stable cycling performance was attributed to synergistic interaction between Ge and carbon and void space that accommodate volume expansion of Ge.

**Figure 10:**
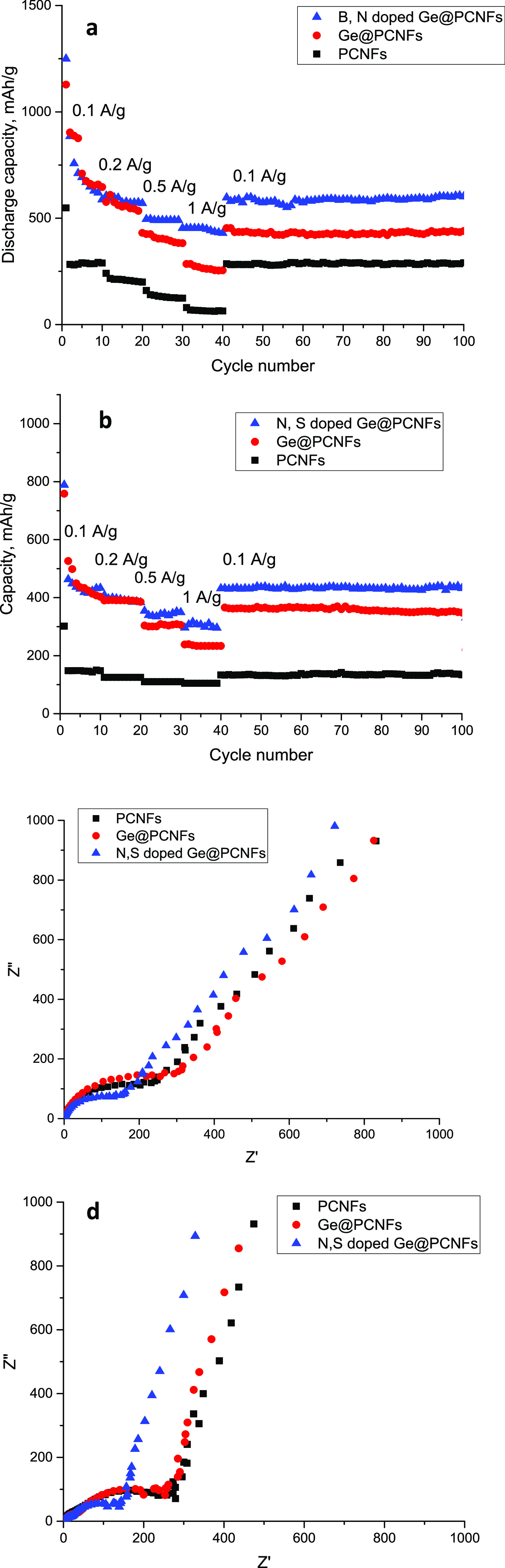
C rate performance (a, b) and EIS spectra (c, d) of N, S-doped Ge@PCNFs in LIBs and (b) SIBs.

C-rate performance of N, S-doped Ge@ PCNFs electrodes in LIBs and SIBs was presented in Figure 10. C rate performance of PCNFs and Ge@ PCNFs was also shown for comparison. N, S-doped Ge@ PCNFs electrodes showed the best performance in both LIBs and SIBs. A capacity of over 450 mAh/g was achieved at 1 A/g while the capacity of Ge@PCNFs was only 255 mAh/g. The result could be attributed to better conductivity due to N, S doping. Moreover, when the current was back to 100 mA/g, N, S-doped Ge@PCNFs exhibited excellent reversibility owing to the enhanced conductivity and active sites. High capacity at high current rates was also reported for electrospun Ge/CNFs, and the result was ascribed to Ge-N chemical bonds that prevent pulverization and create more active sites.^7^

In SIBs, the capacity of N, S-doped Ge@PCNFs was approximately 300 mAh/g at 1 A/g whereas that of Ge@PCNFs was only 236 mAh/g. The capacities for N, S-doped Ge@PCNFs, Ge@PCNFs and PCNFs were 435/407/150, 400/400/112, 357/302/100, and 300/236/106 at 0.1, 0.2, 0.5, and 1 A/g, respectively. Moreover, when the current rate was back to 0.1 A/g, excellent reversibility with a capacity of 432 mAhg was observed from N, S-doped Ge@PCNFs. However, the capacity was only 360 mAh/g at 0.1 A/g for Ge@PCNFs. Liu et al.^35^ deposited Ge and amorphous carbon on porous carbon matrix, and a capacity of around 300 mAh/g was observed in SIBs. High capacity with good cycle performance was seen owing to the amorphous carbon matrix that not only improves conductivity but also prevents pulverization.

EIS spectra of N, S-doped Ge@ PCNFs electrodes for LIBs and SIBs are presented in Figure 10c,d respectively. Owing to enhanced conductivity with N, S doping, N, S-doped Ge@ PCNFs electrodes showed the lowest interfacial resistance compared to all studied electrodes.

As a result, PCNFs with a tubular carbon structure as observed from TEM images buffer a large volume change of Ge during the Li^+^ and Na^+^ insertion and extraction process leading to excellent cycling performance. The appropriate tubular structure increases ion accessibility between active materials and electrolyte and shortens the pathway of ions and electrons, and the conductive carbon networks lead to rapid movement of electrons. Furthermore, N, S-doped Ge@PCNFs act as active sites for ions and give a better electronic conductivity, and thus high capacity is observed even at high rates.

## 4 Conclusions

Centrifugal spinning was used to fabricate binder-free N, S-doped Ge@PCNFs anodes for Li-ion and Na-ion batteries. High reversible capacities of 636 and 443 mAh/g were achieved in lithium-ion and SIBs, respectively. Germanium was homogeneously distributed on PCNFs, and N, S doping further improved the cell kinetics owing to enhanced conductivity and defect sites. The excellent electrochemical results can be attributed to highly dispersed Ge, tubular structured, and interconnected conductive network of centrifugally spun binder-free N, S-doped Ge@PCNFs. The electrochemical results present that combining centrifugal spinning and N, S doping is a promising and facile way to fabricate high-performance electrodes for energy storage systems.

## Supporting Information Available

High-magnification TEM image for N, S-doped Ge@PCNFs and high-resolution scans of the S2p spectrum and N spectrum (PDF)
