# Supplementary material for: Centrifugally Spun Binder-Free N, S-Doped Ge@PCNF Anodes for Li-Ion and Na-Ion Batteries
Source: ACS Omega. 2023 May 3;8(19):16987–95. doi: 10.1021/acsomega.3c00990 (PMC10193401; doi:10.1021/acsomega.3c00990)
Supplement: Supplementary file 1 — ao3c00990_si_001.pdf [file ao3c00990_si_001.pdf]

Supporting Information

Centrifugally spun binder free N, S doped Ge@PCNF anodes for Li ion and Na ion batteries

Meltem Yanilmaz[1,2*], Göktuğ Cihanbeyoğlu[1], Juran Kim[3*]

[1] Nanoscience and Nanoengineering, Istanbul Technical University, Istanbul, Turkey

[2] Department of Textile Engineering, Istanbul Technical University, Istanbul, Turkey

[3]Advanced Textile R&D Department, Korea Institute of Industrial Technology (KITECH), Ansan 15588, Republic of Korea

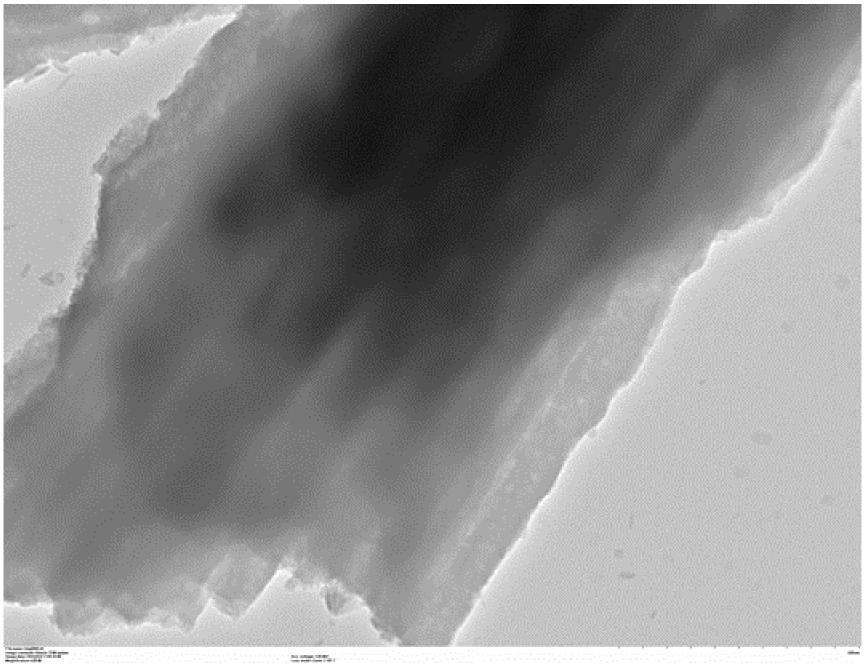

S1 High magnification TEM image for N, S doped Ge@PCNFs

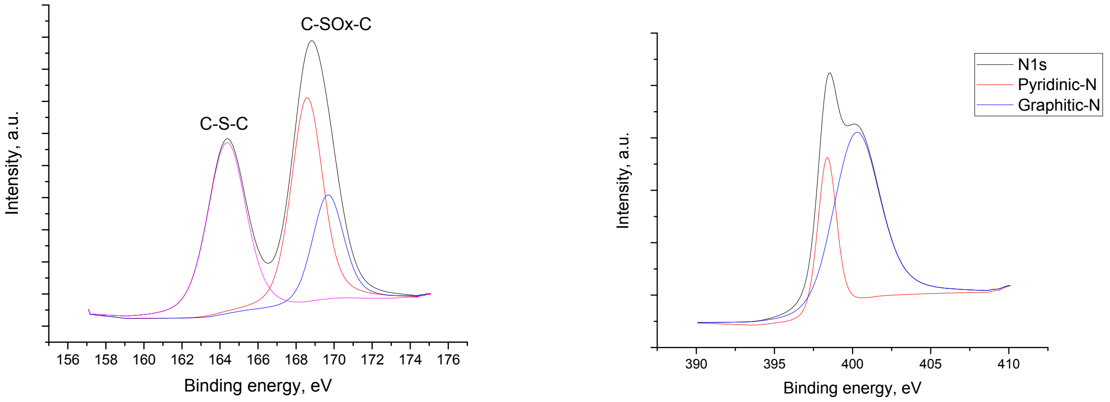

S2 High-resolution scans of S2p spectrum (a) N spectrum (b).
